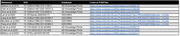# Cellular and molecular changes in a unified and integrated single cell atlas of Alzheimer’s disease across community datasets

**DOI:** 10.1002/alz.087490

**Published:** 2025-01-03

**Authors:** Kyle J Travaglini

**Affiliations:** ^1^ Allen Institute for Brain Science, Seattle, WA USA

## Abstract

**Background:**

Numerous studies have identified AD‐associated molecular and cellular changes to the cortex using single nucleus RNA sequencing (snRNA‐seq) and, to a lesser extent, single nucleus ATAC‐seq (snATAC‐seq), applied to millions of cells across hundreds of donors. It has proven challenging, however, to determine whether changes are consistent because of differences in cohort selection, reported clinical metadata, data pre‐processing, cellular taxonomy construction/mapping, and analytical strategies across studies.

**Method:**

We uniformly re‐processed 10 publicly available datasets (Table 1) that had applied snRNA‐seq to 4.3 million cells from the dorsolateral pre‐frontal cortex (DLPFC) of 780 donors. We integrated each of them with SEA‐AD’s multi‐modal, single cell atlas of AD, which contains 4.5 million cells from the middle temporal gyrus (MTG) and DLPFC that were profiled with snRNA‐seq, snATAC‐seq, snMulitome or spatial transcriptomics (MERFISH). We then harmonized clinical data across donors and mapped all cells to the same 139 fine‐grained cell supertypes in SEA‐AD’s shared MTG and DLPFC cellular taxonomy. With common clinical data and cellular labels, we then compared cohorts and data quality across studies as well as identified cellular changes consistently associated with AD.

**Result:**

Cohorts from nearly every study sampled across the spectrum of plaque and tangle pathology, even in those that performed case‐control analyses. With rare exception, the fraction of donors in each cohort with an APOE4 allele, clinically diagnosed dementia, and severe co‐morbidities were also similar. Comparing datasets, SEA‐AD was uniquely successful in profiling both many donors and many nuclei per donor, while deeply sequencing libraries to drive high gene detection. Most importantly though, of the 34 supertypes that were significantly changed in the DLPFC in SEA‐AD, 8 were also significantly changed across external studies with large enough cohorts to power discovery. This included 5 selectively vulnerable Sst interneuron supertypes and 1 Microglia supertype that were changed early in AD in SEA‐AD.

**Conclusion:**

Consistent and early selective loss of Sst interneurons and increase in disease associated Microglia underscore their importance to AD progression. Our integrated dataset provides a valuable common starting point for future comparative analyses from the community and, we hope, will accelerate discovery of novel therapeutic targets.